# Early and Late Cardiovascular and Metabolic Responses to Mixed Wine: Effect of Drink Temperature

**DOI:** 10.3389/fphys.2018.01334

**Published:** 2018-09-26

**Authors:** Delphine Sarafian, Claire Maufrais, Jean-Pierre Montani

**Affiliations:** Laboratory of Integrative Cardiovascular and Metabolic Physiology, Faculty of Science and Medicine, University of Fribourg, Fribourg, Switzerland

**Keywords:** drink temperature, wine, alcoholemia, hemodynamics, energy expenditure, skin blood flow

## Abstract

**Aim:** Red wine is usually ingested as an unmixed drink. However, mixtures of wine with juices and/or sucrose (mixed wine) are becoming more and more popular and could be ingested at either cold or hot temperature. Although the temperature effects on the cardiovascular system have been described for water and tea, with greater energy expenditure (EE) and lower cardiac workload with a colder drink, little information is available on the impact of temperature of alcoholic beverages on alcoholemia and cardiometabolic parameters. The purpose of the present study was to compare the acute cardiovascular and metabolic changes in response to mixed wine ingested at a cold or at a hot temperature.

**Methods:** In a randomized crossover design, 14 healthy young adults (seven men and seven women) were assigned to cold or hot mixed wine ingestion. Continuous cardiovascular, metabolic, and cutaneous monitoring was performed in a comfortable sitting position during a 30-min baseline and for 120 min after ingesting 400 ml of mixed wine, with the alcohol content adjusted to provide 0.4 g ethanol/kg of body weight and drunk at either cold (3°C) or hot (55°C) temperature. Breath alcohol concentration was measured intermittently throughout the study.

**Results:** Overall, alcoholemia was not altered by drink temperature, with a tendency toward greater values in women compared to men. Early responses to mixed wine ingestion (0–20 min) indicated that cold drink transiently increased mean blood pressure (BP), cardiac vagal tone, and decreased skin blood flow (SkBf) whereas hot drink did not change BP, decreased vagal tone, and increased SkBf. Both cold and hot mixed wine led to increases in EE and reductions in respiratory quotient. Late responses (60–120 min) led to similar cardiovascular and metabolic changes at both drink temperatures.

**Conclusion:** The magnitude and/or the directional change of most of the study variables differed during the first 20 min following ingestion and may be related to drink temperature. By contrast, late changes in cardiometabolic outcomes were similar between cold and hot wine ingestion, underlying the typical effect of alcohol and sugar intake on the cardiovascular system.

## Introduction

Consumption of alcoholic beverages on a daily basis or during festive events can vary broadly in terms of type of drinks and pattern of intake but also with the temperature at which fluids are ingested. In 2015, a Swiss national survey assessing the evolution of attitudes to addictions showed that nearly 60% of alcohol consumed in Switzerland is in the form of wine (Marmet and Gmel, 2016). As a drink, red wine is generally consumed without being mixed or in combination with fruit juice or sugar and can then be drunk either at cold temperature (iced wine “sangria") during summer times or at hot temperature (mulled wine) during winter times. In scientific studies, red wine is usually ingested at ambient temperature but the comparison of the biological effects of a cold *versus* hot alcoholic beverage, to the best of our knowledge, has never been studied in humans.

Previous studies from our lab have examined the cardiovascular and/or metabolic responses to water or tea ingestion at different temperatures. We demonstrated that, compared to body–temperature water, ingestion of 500 ml of cold tap water (3°C) reduced the workload to the heart through vagal tone activation (Girona et al., 2014) and that both cold distilled and tap water increased energy expenditure (EE) by about 3% (Brown et al., 2006; Girona et al., 2014). Recently, we showed that ingestion of 500 ml of Yerba Mate tea served cold (∼3°C), in contrast to hot (∼55°C), decreased heart rate (HR), skin blood flow (SkBf), and increased baroreflex sensitivity (BRS). Furthermore, resting EE increased by 8% with cold tea, suggesting that the rise in thermogenesis is not just related to the cold drink temperature but to the interaction of cold with some of the bioactive ingredients in the tea (Maufrais et al., 2018).

Regarding the acute cardiovascular responses to alcohol, studies have reported that ethanol at relatively moderate doses (0.3–1.0 g/kg body weight) induced transient alterations in the cardiovascular system through changes in cardiac autonomic regulation (van de Borne et al., 1997; Spaak et al., 2008, 2010; Buckman et al., 2015) in healthy normotensive subjects. A recent publication from our lab has shown in healthy young subjects that alcopops (a combination of alcohol and sugar) given at 10°C led to a small decrease in blood pressure (BP), greater decreases in total peripheral resistance (TPR) and BRS, and greater increases in HR and cardiac output (CO), compared with alcohol alone (Maufrais et al., 2017).

Although the impact of drink temperature has been described for common drinks like water and tea, there is a lack of studies in humans addressing the role of the drinking temperature of alcoholic beverages, in particular those made from mixed wine (e.g., “sangria” or “mulled wine”), on cardiovascular and metabolic responses. Our study was designed to test several hypotheses. First, as meal temperature may affect gastric emptying (Sun et al., 1988; Mishima et al., 2009) and thus alcohol absorption and elimination, we tested whether the temperature of mixed wine would affect alcoholemia. Second, as ethanol (Abdel-Rahman et al., 1987) and cold water (Girona et al., 2014) have opposite effects on HR, we tested whether cold mixed wine can offset the tachycardic effects of ethanol and sugar. Finally, as both ethanol and heat are known vasodilators, we tested whether ethanol-induced vasodilation was potentiated by the ingestion of hot beverage. To this aim, we studied in young adults the *interaction of drink temperature* and *alcohol ingestion* on the acute cardiovascular and metabolic responses to mixed wine, consumed either cold (3°C) or hot (55°C).

## Materials and Methods

### Subjects

Fourteen healthy young Caucasian subjects (seven men and seven women) were recruited among students at the University of Fribourg. The mean (± standard deviation) age of the participants was 22.4 ± 2.1 years, weight 63.9 ± 6.7 kg, and body mass index 21.8 ± 2.6 kg/m^2^. We excluded participants with Asian ancestry because of possible intolerance to alcohol, abstainers, chronic drinkers, or those being intolerant to alcohol. All subjects were healthy, weight-stable with no metabolic, cardiovascular, or intestinal diseases. None of the participants was taking drugs, vitamins, or supplements during the previous 7 days that could interfere with the cardiovascular or metabolic control.

Before enrollment in the study, the participants completed a questionnaire regarding their medical history and lifestyle, and familiarized themselves with the experimental procedures and equipment. All participants were instructed to refrain from heavy exercise and from taking caffeinated drinks and alcohol on the day before each experiment. Women were only tested during the follicular phase (days 6–13) of their menstrual cycle. All participants gave written informed consent in accordance with the Declaration of Helsinki. This study was carried out with the approval of the Swiss ethics committee on research involving humans (Canton de Vaud, CER-VD, protocol 2016-01916).

### Study Design

**FIGURE 1:**
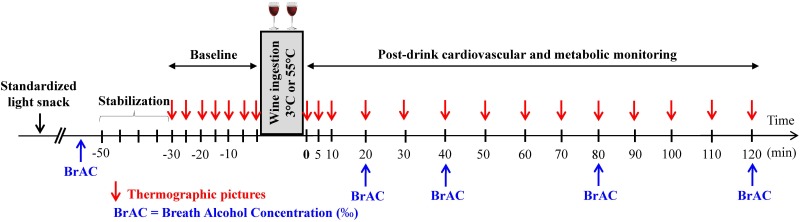
Study design indicating the time at which measurements of breath alcohol concentration (BrAC) and thermographic pictures were done.

The study design is presented in **Figure 1**. All studies took place in an air-conditioned (21–23°C) laboratory dedicated for human metabolic measurements and started at ∼8:30. Every participant attended two sessions (separated at least by 2 days) according to a randomized crossover design. The randomization was performed by using a random sequence generator^1^. On the day of the experiment, to avoid alcohol consumption on an empty stomach, participants were instructed to take at ∼7:30 a light standardized snack provided by us, consisting in one cereal bar (Farmer Soft raspberry, 77 kcal, 13 g carbohydrates, Migros, Switzerland) and 33 cl of ice tea light flavor lemon (33 kcal, 8 g carbohydrates, Migros, Switzerland) providing 110 kcal in total. Upon arrival at the laboratory and after anthropometric measurements (see below), the participants were seated in a comfortable armchair and the cardiovascular monitoring equipment was connected. After reaching cardiovascular and metabolic stability (usually around 15–20 min), a 30-min baseline measurement was conducted. To standardize the pace of drinking, a volume of 400 ml of mixed wine was poured into two thermos glasses of 200 ml each, with instructions to consume each glass over a 4-min period. The drinks were served at either 3 or 55°C. Cardiovascular and metabolic monitoring continued for another 120 min post-drink ingestion. Subjects were permitted to watch neutral documentaries on a flat TV screen and were instructed to relax and avoid movements throughout the measurements.

### Drink Preparation

In a pilot study conducted in our laboratory, we chose the ingredients of the mixed wine after hedonic evaluation by volunteers from our university staff. Both cold and hot drinks were prepared with the same ingredients (red wine, fruit juice, sucrose, distilled water, and spices) at room temperature. The red wine (Viña Albali, Tempranillo 2012, Denner, Switzerland, containing 13% alcohol per volume) was given at 3.84 ml/kg body weight, corresponding to a volume of wine between 204.6 (21.3 g of ethanol) and 286.5 ml (29.8 g of ethanol) for our lightest (53.2 kg) and heaviest (74.5 kg) subjects, respectively, providing 0.4 g ethanol/kg of body weight for all participants. In addition to the red wine, 90 ml of fruit juice (Sarasay Exotic Island, total carbohydrates 15 g/100 ml, Migros, Switzerland), 21.5 g of sucrose, and spices (0.07 g of cloves, 0.3 g of star anise, and 0.6 g of cinnamon) were mixed and diluted in distilled water up to a total volume of 400 ml. The spices were infused (30 min) in the liquids at room temperature under agitation and then the drinks were stored in the fridge 1 day before the test at 2–3°C. On the morning of the test for the hot condition, the mixed wine was hermetically sealed with a cap to prevent loss of liquid by evaporation and maintained into a water bath set at 55°C for 15–20 min to reach the set temperature. At this temperature, no change in volume was observed. Changes in volume or alcohol content are not expected since the boiling temperature of pure ethanol is higher than 75°C at the altitude of Fribourg (630 m) and is even higher when mixed with water. For the cold condition, the drink was taken out of the refrigerator a few minutes before ingestion and its temperature was checked.

### Anthropometric and Body Composition Measurements

Prior to testing and after the subjects voided their bladder, body weight and height were measured using a mechanical column scale with integrated stadiometer (Seca model 709, Hamburg, Germany) to the nearest 0.1 kg and 1 mm, respectively. Body composition [body mass index, body fat mass (FM), fat-free mass (FFM), and total body water] was assessed non-invasively by multi-frequency bioelectrical impedance analysis (Inbody 720, Biospace Co., Ltd., Seoul, Korea) on the preliminary visit and on each testing day.

### Cardiovascular Recordings

Cardiovascular recordings were performed non-invasively using the Task Force Monitor (CNSystems Medizintechnik, Graz, Austria). Cardiac intervals were recorded continuously by electrocardiography to compute HR. Stroke volume (SV) was assessed by impedance cardiography as previously described (Maufrais et al., 2018). Continuous BP was recorded from either the index or middle finger of the left hand every 10 min and was automatically calibrated to oscillometric brachial BP on the contralateral arm measured every 5 min. The left hand with the continuous BP cuff rested on a comfortable cushion at heart level on a height adjustable table. CO and TPR were then determined by calculation from SV, HR, and oscillometric brachial BP signals. High frequency (HF: 0.15–0.40 Hz) power components of RR intervals (HF_RRI) were assessed to have an index of parasympathetic activity (Pagani et al., 1997). Data given in absolute values (ms^2^) were analyzed after natural logarithmic transformation (HF_RRI_LN). BRS was determined from spontaneous fluctuations in BP and cardiac interval using the sequence method (Bertinieri et al., 1985).

### Metabolic Recordings

Energy expenditure and respiratory quotient (RQ) (both derived from oxygen consumption and carbon dioxide production) were measured non-invasively and continuously using a ventilated hood indirect calorimeter (Quark RMR, Cosmed, Rome, Italy) as described previously (Miles-Chan et al., 2015). After an initial resting period of 15–20 min to allow gas exchange values to reach a steady state, resting EE was measured over a 30-min baseline period. The hood was removed during wine ingestion and then placed back over the subjects for a further 120 min after the drink. During breath alcohol concentration (BrAC) measurements, the hood was not removed but slightly shifted up to allow BrAC measurement. Metabolic data during and 1 min following breath alcohol measurement were excluded from analysis to avoid artifacts and allow the re-equilibrium of gases in the hood.

### Cutaneous Blood Flow and Skin Temperature

Skin blood flow was assessed continuously on the back on the right hand by laser speckle contrast imager (LSCI) (PeriCam PSI System, Perimed). The head of the LSCI was placed at 12 cm above the skin, with a defined region of interest of 4 × 4 cm area of skin for all subjects. Routine verification for LSCI was made with the calibration box supplied with the device according to the procedure described by the manufacturer. Skin hand temperature was assessed by the infrared camera FLIR E6 (FLIR Systems) which was positioned on a fixed adjustable support and placed at a distance of 25 cm from the subject’s hand. Three thermographic pictures of the right hand were made at different time points (**Figure 1**). Thermic pictures were analyzed at specific regions of interest as described previously (Maufrais et al., 2017) using the FLIR Tools software (version 6.1, FLIR Systems).

### Breath Alcohol Concentration

As BrAC correlates highly with the concentration of alcohol in the blood (Jones and Andersson, 2003; Hey and Haslund-Vinding, 2006), we used a breath ethylometer (Model 6820, Dräger SA, Germany) to estimate alcoholemia without the stress of blood sampling, at various time points, at baseline and at 20, 40, 80, and 120 min post-drink. Each subject made a prolonged exhalation for 5 s into the tube of the alcotest and duplicate measurements were made. Average of these two recorded values were used for the analysis. At the end of the experiment, subjects were given a small snack and BrAC was determined to ensure that the subjects left the lab well below the Swiss legal limit of 0.5‰.

### Visual Analog Scale

Participants’ subjective ratings of perceived sensations after alcohol ingestion (early and late thermal sensations, comfort, and drunkenness) were done at the end of the experiment by completing three items on a visual analog scale (VAS). The scale consisted in a continuous horizontal line that measured 10 cm in length and was anchored by words that described extremes sensations (Hayes and Patterson, 1921). The scale ranged from 0 on the left (very cold, not comfortable at all, and not drunk at all) to 10 on the right (very hot, extremely comfortable, and extremely drunk). Participants completed the scales by placing a single vertical line through each horizontal line and VAS score was calculated by measuring, to the nearest millimeter, the distance from the left edge to the mark placed by the subjects along the scale. VAS were presented to the participants at the very end of the test and only 10 of 14 VAS were collected because its use was introduced into the protocol after the study had begun.

### Data Processing

Cardiovascular, metabolic, and cutaneous variables were first processed per minute and averaged over 5 min. Then, data were averaged over 15 min intervals during the baseline pre-drink period. After drink ingestion, data were averaged every 10 min for the first 20 min, and then every 20 min until the end of test. For all the variables, the changes from baseline were calculated as the absolute values averaged over 10 and 20 min intervals from which was subtracted the 30 min of baseline measurement, and are presented as deltas (Δ). Relative to baseline, early responses were averaged over 0–20 min and late responses over 60–120 min post-drink and are presented as deltas (i.e., average of absolute between 0–20 and 60–120 min post-drink periods, respectively, minus the average over the 30-min baseline period). CO was computed as the product of SV and HR. Mean arterial BP (MBP) was calculated from brachial diastolic BP (DBP) and systolic BP (SBP) measured from the right arm as follow: MBP = DBP + 1/3(SBP-DBP). TPR was calculated as MBP/CO. Double product (DP) was calculated as the product of HR and SBP and provides valuable information about the oxygen consumption of the myocardium (van Vliet and Montani, 1999). Cardiac power output (CPO) was calculated as MBP × CO/451 (Fincke et al., 2004). Mean BrAC between 20 and 120 min post-ingestion (BrAC_[20-120]__min_) was calculated as the area under the concentration–time curve (AUC) divided by 100 min, with AUC computed by the trapezoid method.

### Statistical Analysis

All values are reported as mean ± SEM unless otherwise specified. Statistical analysis was performed using statistical programs: (i) Statistix version 8.0, Analytical Software, St. Paul, MN, United States and (ii) GraphPad Prism, version 7, GraphPad software, Inc., La Jolla, CA, United States. Testing for normal distribution was performed using D’Agostino and Pearson Omnibus normality test. Statistical analysis was performed by two-way ANOVA for repeated measures with time and temperature (cold and hot) as within-subject factors. When significant differences were found, the effects of each drink temperature over time were analyzed by comparing values at each time point over the post-drink period with the basal values recorded before drinking. Dunnett’s multiple comparison *post hoc* testing was used to test for changes over time from baseline levels. Difference in BrAC data between drink conditions was tested by using Student’s paired *t*-test. Sex differences in BrAC for each drink condition were assessed by Student’s unpaired *t*-test. For all tests, significance was set at *p* < 0.05 (two-tailed). Pearson and Spearman correlation analysis were used to determine the association between mean BrAC_[20-120]__min_ and body composition measurements.

## Results

### Breath Alcohol Concentration and Correlation With Body Composition

**FIGURE 2:**
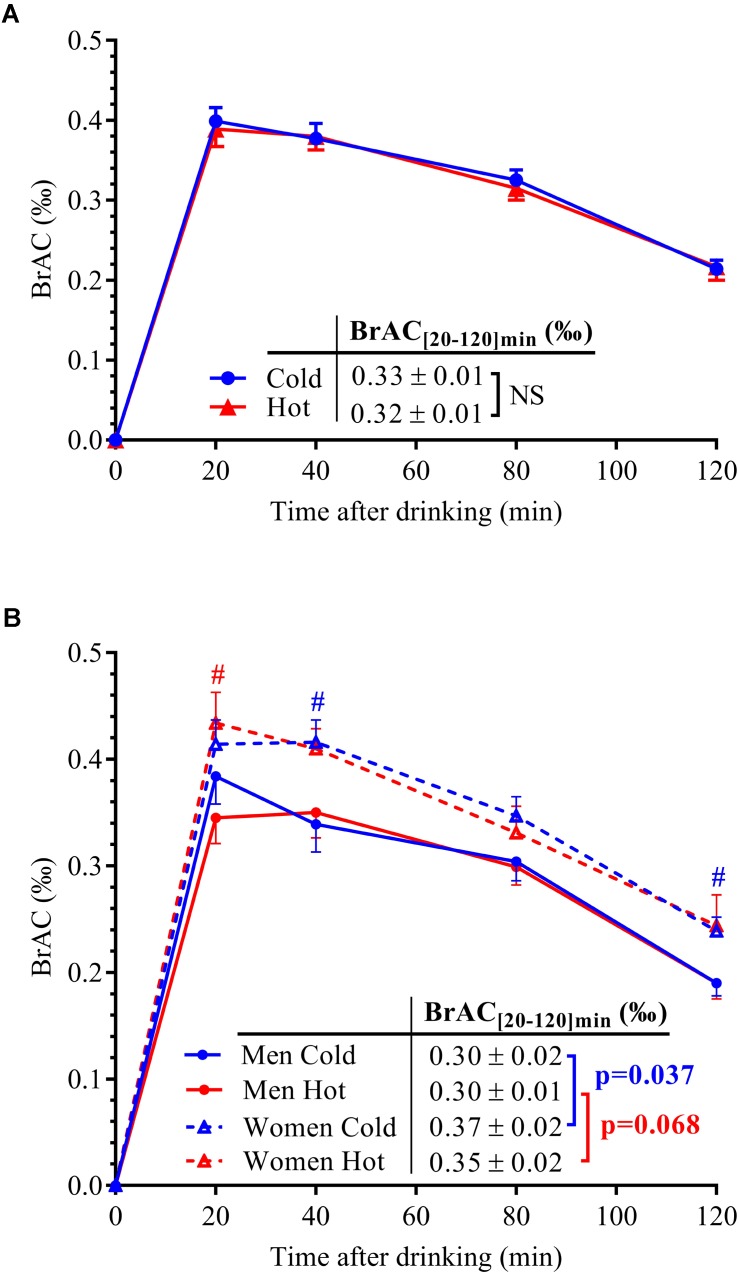
**(A)** BrAC over time after alcohol ingestion in the 14 subjects. **(B)** BrAC data were separated by sex (seven men: solid lines and seven women: dotted lines) for both drink temperatures of ingestion (blue lines: cold wine; red lines: hot wine). Area under the concentration–time curve for BrAC between 20 and 120 min post-drink ingestion, i.e., mean BrAC_[20-120]__min_ (‰) was indicated according to sex and drink conditions, with corresponding *p*-values computed by unpaired *t*-test. All data are mean ± SEM. #*p* < 0.05 significant difference between men and women (unpaired *t*-tests).

Time course of mean BrAC (‰) after cold and hot wine ingestion in 14 subjects is presented in **Figure 2**. Firstly, the time to peak ethanol concentration was similar after both drink temperatures and was attained after 20 min post-ingestion. BrAC_[20-120]__min_ were similar after cold (0.33 ± 0.01‰) and hot (0.32 ± 0.01‰; *p* = 0.68) wine ingestion when pooling all 14 subjects (**Figure 2A**) or when analyzing results from men or women separately. However, when separating data by sex, BrAC_[20-120]__min_ was significantly higher in women compared to men after cold wine (0.37 ± 0.02‰ vs. 0.30 ± 0.02‰, respectively; *p* = 0.037), with a tendency toward greater values in women in response to hot wine (0.35 ± 0.02‰ vs. 0.30 ± 0.01‰; *p* = 0.068; **Figure 2B**). Assessment of body composition in our participants revealed that compared to men, most of the women have lower FFM, whether those parameters were expressed in absolute (*p* < 0.0001) or when adjusted by body weight (*p* < 0.01). In addition, women had higher body FM and percent body fat (*p* < 0.05 and *p* < 0.01, respectively) than men.

**FIGURE 3:**
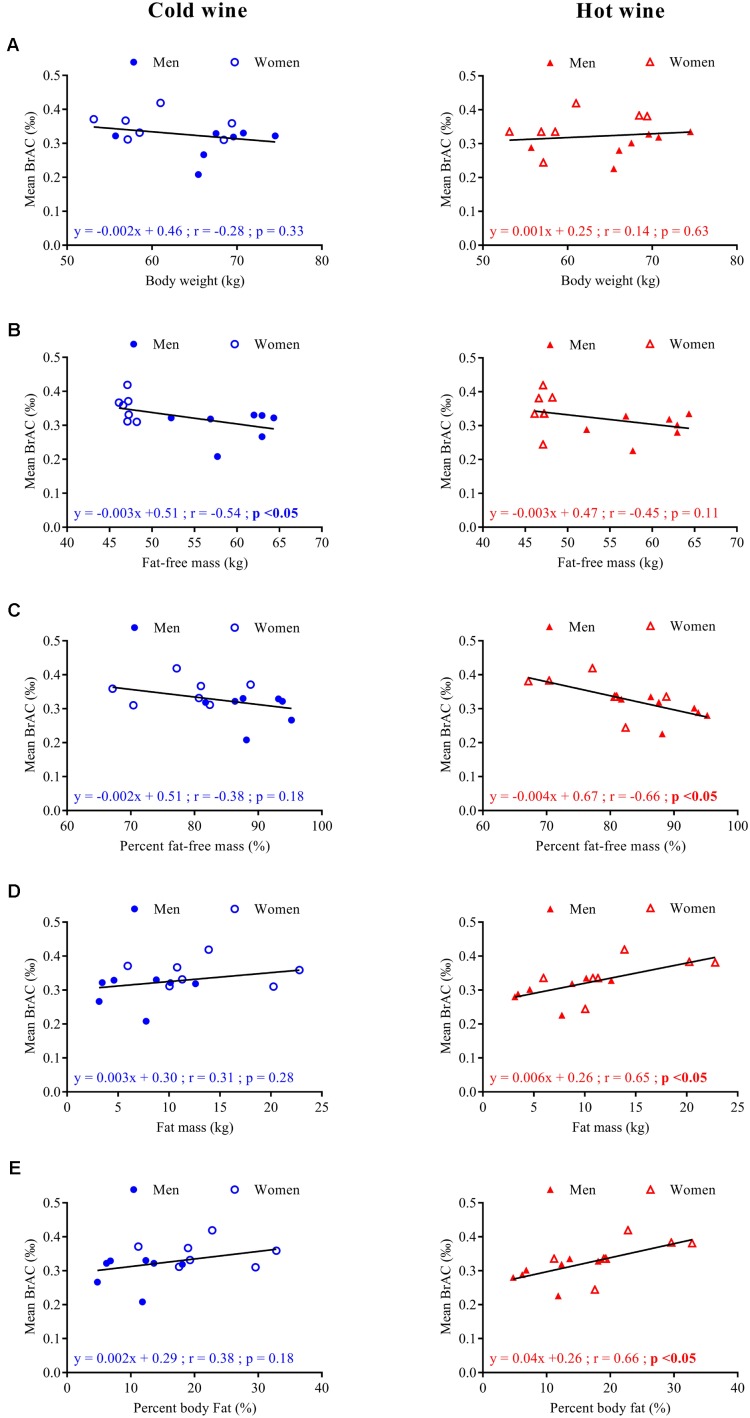
Correlation analysis for seven men (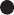, 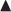) and seven women (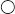, Δ) between mean BrAC_[20-120]__min_ after cold (left panels, blue symbols) and hot (right panels, red symbols) wine ingestion and body weight **(A)**, fat-free mass in kg **(B)**, percent fat-free mass **(C)**, fat mass in kg **(D)**, and percent body fat **(E)**. Regression lines (—), equations of the linear regressions, correlation coefficients (*r*), and *p*-values were obtained for 14 subjects.

Correlation analyses between mean BrAC over the 20–120 min post-drink period and parameters of body composition are presented in **Figure 3**. No correlation was found between BrAC and body weight in either drink condition. However, BrAC_[20-120]__min_ tended to be negatively correlated with FFM and percent FFM, and seemed positively associated with FM and percent body fat.

### Cardiovascular Responses

**FIGURE 4:**
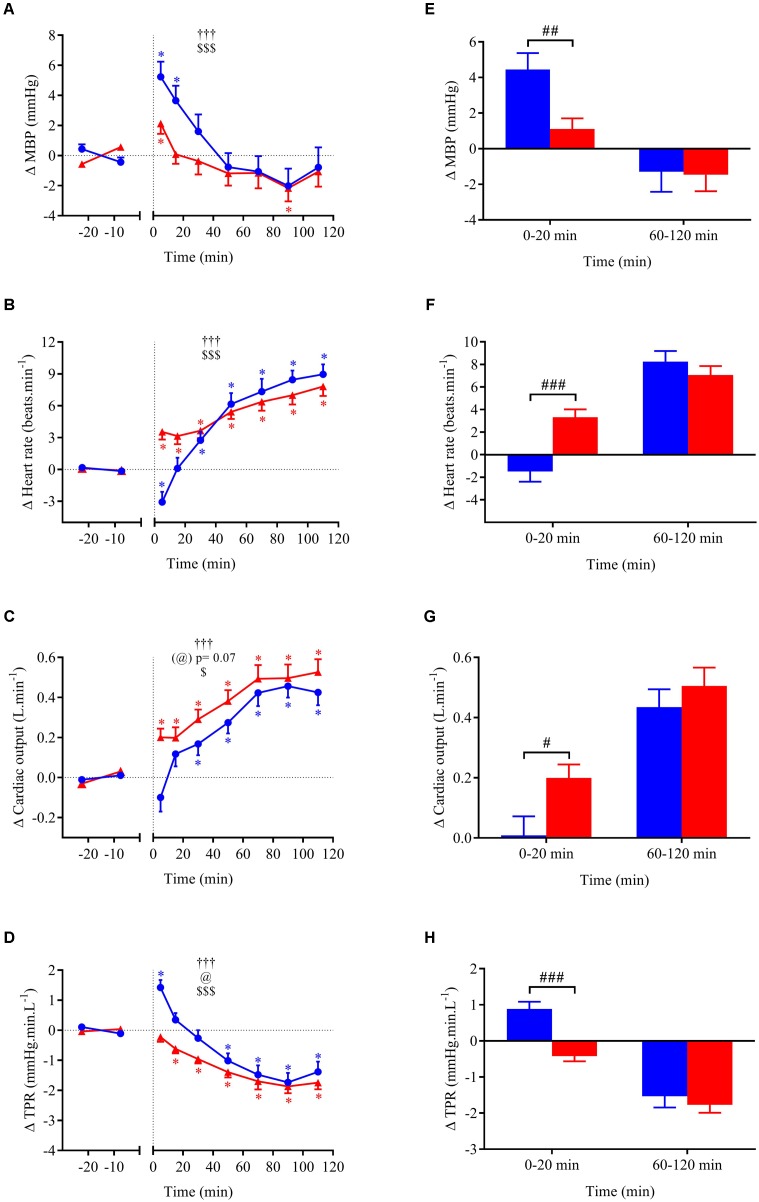
**(A–D)** Time course of the changes in MBP, heart rate (HR), cardiac output (CO), and total peripheral resistance (TPR) presented as delta (i.e., absolute changes relative to baseline levels). **(E–H)** Mean responses averaged over 0–20 and 60–120 min post-drink periods presented as delta (i.e., average over 0–20 and 60–120 min post-drink minus the average over the 30-min baseline period, respectively). Drinks: cold wine (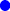, 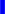); hot wine (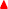, 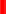). Values are mean ± SEM. Symbols for ANOVA analysis: †, time effect; @, temperature effect; and $, time × temperature interaction effects. ^∗^Significant difference over time from baseline values. ^#^Significant difference between drink conditions (cold vs. hot). The level of significance for all symbols was mentioned as follows: one symbol (*p* < 0.05), two symbols (*p* < 0.01), and three symbols (*p* < 0.001).

**FIGURE 5:**
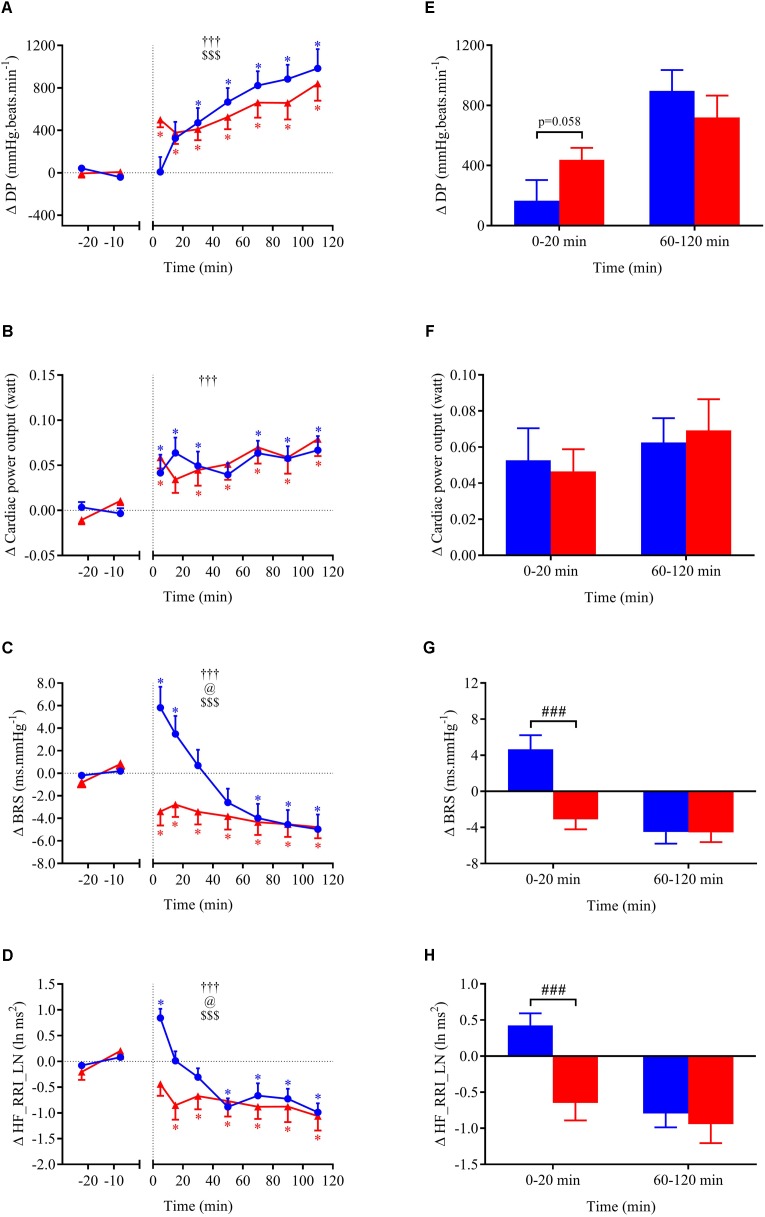
**(A–D)** Time course of the changes in double product (DP), cardiac power output (CPO), baroreflex sensitivity (BRS), and high-frequency power components of RR intervals (HF_RRI_LN) presented as delta (i.e., absolute changes relative to baseline levels). **(E–H)** Mean responses averaged over 0–20 and 60–120 min post-drink periods presented as delta (i.e., average over 0–20 and 60–120 min post-drink minus the average over the 30-min baseline period, respectively). Drinks: cold wine (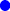, 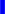); hot wine (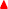, 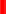). Values are mean ± SEM. Symbols for ANOVA analysis: †, time effect; @, temperature effect; and $, time × temperature interaction effects. See legend of **Figure 4** for level of significance.

**FIGURE 6:**
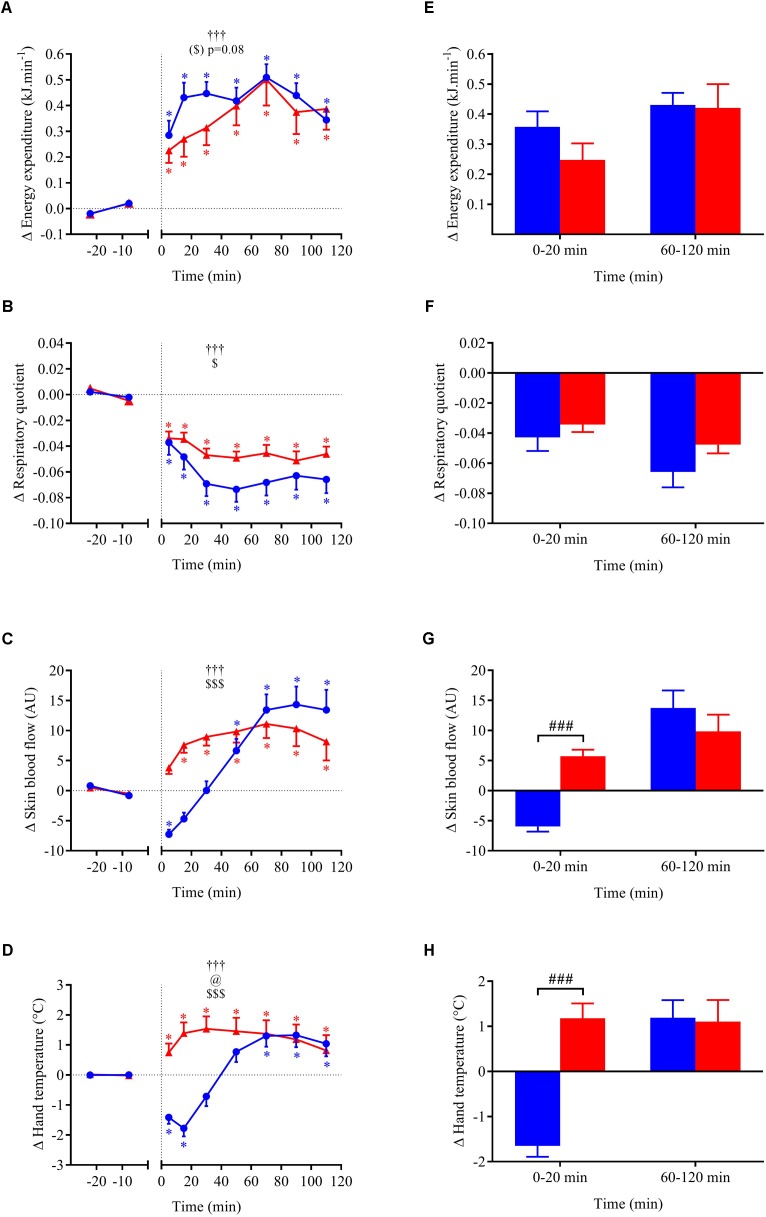
**(A–D)** Time course of the changes in energy expenditure, respiratory quotient, skin blood flow, and hand skin temperature presented as delta (i.e., absolute changes relative to baseline levels). **(E–H)** Mean responses averaged over 0–20 and 60–120 min post-drink periods presented as delta (i.e., average over 0–20 and 60–120 min post-drink minus the average over the 30-min baseline period, respectively). Drinks: cold wine (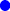, 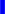); hot wine (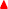, 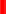). Values are mean ± SEM. Symbols for ANOVA analysis: †, time effect; @, temperature effect; and $, time × temperature interaction effects. See legend of **Figure 4** for level of significance.

Cardiovascular, metabolic, and thermic parameters at baseline are summarized in **Table 1** and were similar between the two experimental sessions. Time course of changes (left panels A–D) and mean changes over 0–20 min and 60–120 min post-drink periods (right panels E–H) for cardiovascular and metabolic variables are shown in **Figures 4A–H**, **5A–H**, **6A–H**. Ingestion of mixed wine at two different temperatures (cold and hot) resulted in significant interaction effects (time × wine temperature, *p* < 0.05) for all cardiovascular parameters except for CPO and EE. The differences due to drink temperature were mostly seen during the early period (0–20 min) whereas the late responses (60–120 min) were similar between the two conditions.

**Table 1:** Baseline cardiovascular and metabolic data recorded before wine ingestion in 14 subjects.

	Cold wine	Hot wine	*p*-value
SBP (mmHg)	108.0 ± 2.4	105.8 ± 1.7	NS
MBP (mmHg)	80.8 ± 1.9	79.1 ± 1.6	NS
DBP (mmHg)	67.4 ± 1.9	65.9 ± 1.6	NS
Heart rate (beats min^-1^)	62.1 ± 1.9	62.3 ± 3.0	NS
DP (mmHg beats min^-1^)	6695 ± 216	6578 ± 311	NS
Stroke volume (ml)	82.1 ± 2.5	81.4 ± 1.9	NS
Cardiac output (l min^-1^)	5.07 ± 0.16	5.03 ± 0.18	NS
TPR (mmHg min l^-1^)	16.2 ± 0.7	16.0 ± 0.6	NS
Cardiac power output (watt)	0.91 ± 0.03	0.88 ± 0.04	NS
Baroreflex sensitivity (ms mmHg^-1^)	23.7 ± 2.0	23.1 ± 2.1	NS
HF_RRI_LN (ln ms^2^)	6.59 ± 0.25	6.76 ± 0.28	NS
Energy expenditure (kJ min^-1^)	4.55 ± 0.15	4.55 ± 0.15	NS
Respiratory quotient	0.85 ± 0.01	0.83 ± 0.01	NS
Skin blood flow (AU)	40.1 ± 1.5	42.8 ± 2.2	NS
Hand temperature (°C)	34.0 ± 0.4	33.7 ± 0.5	NS

*Data are mean ± SEM. Systolic blood pressure (SBP), mean blood pressure (MBP), diastolic blood pressure (DBP), double product (DP), total peripheral resistance (TPR), and natural logarithmic transformation of high-frequency power components of RR intervals (HF_RRI_LN). NS, not significant; AU, arbitrary units.*

**FIGURE 7:**
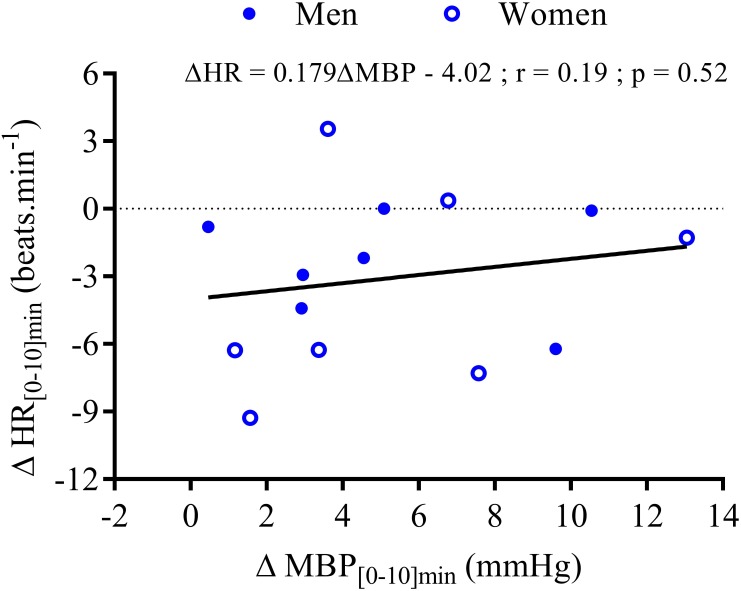
Correlation analysis between the changes in mean HR and the changes in MBP during the first 10 min after wine ingestion for seven men (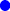) and seven women (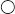). The equation of the linear regression, coefficient correlation (*r*), and *p*-value are provided for 14 subjects.

Within the first 20 min post-drink, MBP was transiently increased after cold wine compared to hot wine (+4.4 ± 0.9 vs. +1.1 ± 0.6 mmHg, respectively; *p* < 0.01, **Figure 4E**). Then, MBP returned around baseline values for both drink conditions. In the first 10 min after cold ingestion, we observed a significant short-lasting drop in HR below baseline (-3.1 ± 1 beats min^-1^; *p* < 0.05), while HR instantly increased after hot wine (+3.5 ± 0.7 beats min^-1^; *p* < 0.05). Twenty minutes after ingestion of either drink temperature, HR gradually increased above baseline levels and remained elevated until the end of test. Correlation analysis indicated no association between changes in MBP and changes in HR over the first 0–10 min after cold wine (*r* = 0.19, *p* = 0.52; **Figure 7**). In addition, the changes in HR were not correlated to subjects’ body weight for both drink conditions. The time course of the changes in CO was very similar to the one of HR, with a sustained increase during the late phase. In the first 10 min after cold wine ingestion, TPR showed an initial rise above baseline (+1.42 ± 0.25 mmHg min l^-1^; *p* < 0.05) while TPR was unchanged with hot wine. Over time, systemic resistance fell below baseline levels for both drink conditions but the decline in TPR was detected earlier with hot wine (*t* = 20 min) than with cold wine (*t* = 50 min) (**Figure 4D**). Both drink temperatures increased DP and CPO above baseline, but DP tended to be more elevated with hot wine over 0–20 min average (*p* = 0.058; **Figure 5E**). Autonomic responses differed significantly between drink conditions (*p* < 0.001) within the first 20 min following wine ingestion but not in the second hour (**Figures 5G,H**). Shortly after drinking hot wine, BRS and HF_RRI_LN significantly dropped below baseline levels and remained low during the entire post-drink period. On the contrary, BRS and HF_RRI_LN were both increased in the first 20 min after cold wine (**Figures 5C,D**) and then, progressively decreased below baseline, reaching values of the hot condition.

### Metabolic and Thermic Responses

For metabolic and cutaneous parameters, the changes over time in response to wine ingestion are shown in **Figure 6**. Both cold and hot wine ingestion led to a significant increase in EE above baseline (*p* < 0.05) with a tendency for EE to be more elevated in the early phase with cold temperature (ANOVA temperature effect, *p* = 0.08) compared to hot (+7.9% vs. 5.3%, respectively; panel E), but not during the second hour of the test. Regarding early perceived thermal sensations just after drink ingestion, participants felt cooler after drinking cold wine whereas they felt warmer after hot wine (VAS score 4.3 ± 0.8 cm vs. 7.4 ± 0.5 cm, respectively; *p* = 0.008). Immediately after cold wine ingestion, 6 out of 10 subjects felt a sensation of cold that was alleviated by a blanket that was made available for thermal comfort. Both drink temperatures led to a significant decrease in RQ relative to baseline (*p* < 0.05). However, the drop in RQ seemed more pronounced with cold wine than hot wine (overall mean 0–120 min = -0.060 ± 0.009 vs. -0.046 ± 0.004, respectively; *p* = 0.12) but no statistical difference was found. Time course of the early changes in SkBf and hand temperature differed according to the temperature of the drinks. SkBf and hand temperature were initially and significantly reduced after cold wine whereas they were augmented with hot wine (*p* < 0.001; panels G and H). After 30 min post-ingestion, we observed a subsequent rise in those parameters with both drink temperatures.

## Discussion

Considering the lack of *in vivo* studies comparing the effects of a cold *versus* a hot alcoholic beverage on cardiovascular and metabolic responses, it was important to test the impact of drink temperature on alcoholemia and on the cardiometabolic responses to alcohol. Our major findings showed that (1) drink temperature of mixed wine had no effect on alcoholemia; (2) cardiometabolic responses to mixed wine mostly differed in the early phase following ingestion. By contrast, late responses were similar between cold and hot mixed wine ingestion, underlying the typical effect of alcohol and sugar intake on the cardiovascular system; (3) consuming alcohol at a hot temperature seemed to potentiate vascular effects as it induces early vasodilation and rapidly increases SkBf and hand temperature.

### Alcohol Absorption in Response to Cold vs. Hot Wine Ingestion

Since alcohol absorption and elimination could be dependent on gastric emptying rate (Holt et al., 1980), we tested whether the drinking temperature would influence BrAC responses. As shown in **Figure 2**, peak BrAC at *t* = 20 min post-ingestion and time course of BrAC values (*n* = 14; panel A) were similar after ingestion of both cold and hot mixed wine, suggesting that the temperature at which the alcohol was ingested did not influence alcohol absorption. Our findings are in line with a study (McArthur and Feldman, 1989) which reported that 360 ml of hot (58°C), body temperature (37°C), and cold (4°C) coffee infused in the stomach had similar effects on gastric emptying. Although the effects of meal and drink temperatures on gastric emptying have been studied by others (Sun et al., 1988; Mishima et al., 2009), conflicting results still exist.

The pharmacokinetics of ethanol, i.e., BrAC profile over time, was similar for both cold and hot wine temperatures as well as for each sex group indicating that the temperature of mixed wine has no effect on alcoholemia. Moreover, women tended to have higher BrAC levels in response to alcohol compared to men, as previously reported (Frezza et al., 1990); we showed here that this finding was independent of drink temperature. Ethanol is distributed in free body water which represents around 73% of FFM (Fuller et al., 1992), the solubility in fat and bones being negligible. Because ethanol was given per kg of body weight in our study and most women had a lower percent FFM than men, this could explain the tendency for the correlation between BrAC and FFM, and why women have higher BrAC levels than men. However, body composition did not fully explain BrAC responses. Rather, alcoholemia can also be influenced by sex differences in gastric aldehyde dehydrogenase activity and first-pass metabolism, both of which are reduced in women (Frezza et al., 1990; Baraona et al., 2001).

### Early Temperature-Induced Cardiovascular and Cutaneous Responses

Based on our results, we can distinguish early and late cardiometabolic changes in response to different temperatures of wine ingestion. As thermosensitive receptors have been described in the gastrointestinal tract of man (Villanova et al., 1997), thermal stimuli from cold and hot wine could initiate early temperature-induced cardiovascular changes until beverage temperature equilibrate to body temperature. In previous studies, the equilibrium of beverage’s temperature in the body was achieved 15–30 min after ingesting cold or warm drinks of equivalent volumes (Sun et al., 1988; McArthur and Feldman, 1989). Interestingly, most cardiovascular changes observed in our study between cold and hot wine were detected within this period, and coincided with peak BrAC (*t* = 20 min).

The initial temperature-related decrease in HR, and increases in TPR and MBP after cold wine are consistent with previous results from our lab showing similar changes in those variables after drinking alcopops (vodka with sugar, 400 ml at 10°C) (Maufrais et al., 2017). Since we did not observe any association between mean changes in HR and changes in MBP over 10 min post-drink, the early HR decrease after cold wine could be the result of temperature-induced direct vagal reflex responses rather than baroreflex activation related to the initial increase in MBP. The immediate reduction in SkBf and skin hand temperature observed in the extremities could be related to the activation of thermoreceptors (temperature-sensitive afferent neurons) in the upper gastrointestinal tract induced by internal cooling after cold drink ingestion. Indeed, evidence of visceral thermoreceptors in humans (Morris et al., 2017) and opposite gastric reflexes induced by cold and hot stimuli, respectively (Villanova et al., 1997), support the potential role of these thermoreceptors in the modulation of HR and vasomotor activity by fluid temperature.

By contrast, hot wine caused an immediate rise in HR that was accompanied by a constant decrease in parasympathetic activity indices (BRS and HF_RRI_LN) throughout the test. Heat-activated thermoreceptors seemed to have different effects from cold temperature on the cardiovascular system, since visceral warming (fluid at 45°C) caused a gastric thermoreflex in rats (tachycardia, hypotension) (Rozsa et al., 1988). As opposed to cold wine, the early decrease in TPR and the parallel increase in SkBf observed after hot wine ingestion suggest an early and sustained peripheral vasodilation, which can explain the steady increase in skin temperature during the whole test. Therefore, hot temperature of alcohol seems to accelerate and exacerbate the peripheral vasodilator effect of red wine (Barden et al., 2013). It is worth pointing out that the physical and chemical properties of our wine should not have been altered by moderate heating (55°C) since wine heated at much higher temperature (75 and 125°C) preserved its *in vitro* vasodilator activity in the isolated rat and guinea pig aorta compared to wine without thermal stress (Mudnic et al., 2011).

### Late Alcohol-Induced Cardiovascular Responses

The cardiovascular changes observed after drinking cold or hot mixed wine were similar for all variables measured in the late phase of ingestion (60–120 min). These late changes are in accordance with previous studies exploring the acute effects of alcohol, although the temperature of alcohol is rarely mentioned in the literature and assumed to be at room temperature. After 20 min post-ingestion, when the ingested liquids should have reached body thermal equilibrium (McArthur and Feldman, 1989), all the variables measured in our study followed the same trend, regardless of initial drink temperature.

Alcohol, particularly in red wine, is known to have vasodilator effects in both normotensive and hypertensive individuals (Porteri et al., 2010; Barden et al., 2013). While ingestion of mixed wine caused a decrease in peripheral resistance, there was little impact on MBP, which could be explained by increases in HR and CO. Many studies on alcohol at similar or higher doses also do not report changes in BP (Abdel-Rahman et al., 1987; van de Borne et al., 1997; Spaak et al., 2008), whereas others showed a direct pressor effect of alcohol (Grassi et al., 1989; Iwase et al., 1995). Our results showing a decrease in HR variability and BRS are consistent with other studies reporting diminished BRS and cardiac vagal tone indices (Abdel-Rahman et al., 1987; Koskinen et al., 1994; Reed et al., 1999; Levanon et al., 2002; Spaak et al., 2010; Buckman et al., 2015). Lastly, the capacity of alcohol in vasodilating and stimulating thermogenesis may contribute to the elevated SkBf and skin hand temperature seen in our study.

Aside from the effects of alcohol, we should also consider the contributory effects of the carbohydrates that were added to the wine (35 g of carbohydrates as fruit juice and sucrose), since we can neglect the total amount of oligosaccharides in the Tempranillo red wine itself, which ranges from 0.075 to 0.3 g/l (Quijada-Morin et al., 2014). Carbohydrate administration could stimulate the sympathetic nervous system activity, with insulin being a potential mediator for this activation (Young and Landsberg, 1982). A previous study from our lab indicated that ingestion of water with sucrose (60 g) at 22°C increased CO, decreased TPR but had no impact on MBP (Grasser et al., 2014). We have also observed that compared to vodka, the combination of vodka (given at 0.4 g ethanol/kg) and sucrose (48 g) induced a greater decrease in MBP and TPR as well as a greater increase in CO, HR, SkBf, and hand temperature (Maufrais et al., 2017), underlying the additive effect of carbohydrates mixed with alcohol on the measured parameters.

### Metabolic Responses to Cold Versus Hot Wine Ingestion

In our study, both drink temperatures increased resting EE with a tendency toward a more pronounced effect with cold (+7.9%) than hot wine (+5.3%) in the first 30 min after ingestion. Consistent with previous studies from our laboratory (Brown et al., 2006; Girona et al., 2014), there is a trend for cold drinks to increase resting EE to higher levels, maybe contributing to warming the cold fluid ingested. Compared to room- or body-temperature water, resting EE was reported to be increased by less than 5% after ingestion of cold (3°C) distilled water (Brown et al., 2006) or tap water (Girona et al., 2014). Unlike water, which is calorie-free and has a small thermogenic effect (Charriere et al., 2015), the higher EE increase in response to mixed wine is possibly related to the thermogenesis of ethanol and sugars contained in our drink. The theoretical energy required to warm cold water from 3 to 37°C would be 57 kJ (400 ml × 4.186 J/g°C × 34°C). Despite the presence of these energetic substrates (alcohol and sugars), the total increase in EE during the first 30 min after cold wine was only ∼12 kJ, which is much lower than the energy cost of warming the cold fluid. Therefore, the warming of cold wine seems more related to heat preservation due to thermoregulatory reflex mechanisms leading to peripheral skin vasoconstriction to avoid heat-loss. This assumption is consistent with the lower SkBf seen in our study in subjects after drinking cold wine, but not hot wine. This is in accordance with a study where compared to a fluid at 37°C, ice slurry ingestion led to important reductions of heat loss from the skin and greater heat storage during exercise in the heat (Morris et al., 2016).

As previously described (Schutz, 2000), ethanol *per se* has also a thermogenic effect since its energy density is 7.1 kcal/g of ethanol. Small doses of alcohol in the form of red wine have been shown to increase oxygen consumption (Rosenberg and Durnin, 1978) and to enhance resting metabolic rate in social drinkers (Klesges et al., 1994). Using the ventilated hood system, the acute intake of alcohol at similar doses (20–30 g) was shown to enhance metabolic rate by 4–7% over baseline in fasted subjects (Weststrate et al., 1990; Suter et al., 1994), which is in the range of our results. In addition to ethanol, the caloric load of carbohydrates added to the wine can contribute to the increase in thermogenesis, as stated elsewhere (Welle et al., 1981; Acheson et al., 1984; Jequier, 1984).

Regarding substrate oxidation, the interpretation of our results in relation to ethanol intake must be taken with caution. Similar drops in RQ were observed after drinking beer (Esteban-Fernandez et al., 2014) and alcohol ingested at different concentrations increased O_2_ consumption but not CO_2_ production, leading to a lower RQ (Weststrate et al., 1990). However, this lesser RQ in response to alcohol does not necessarily indicate an increase in lipid oxidation since ethanol oxidizes with a RQ around 0.66 (Schutz, 2000).

## Conclusion

We provided here a comprehensive comparison of a heated vs. cooled alcoholic beverage effects on the cardiovascular system and metabolism in the same healthy volunteers in a randomized crossover design. The new findings reside in the fact that the temperature of wine consumed had no influence on the time course of BrAC in either sex. Women presented, however, higher alcohol levels than men, which could be partly explained by difference in body composition. The drinking temperature altered mostly cardiovascular responses in a time-dependent fashion, with fluid temperature no longer playing a role in the late responses. Within the first 20 min after alcohol ingestion, we identified differing cardiovascular responses that may be related to drink temperature, with cold mixed wine inducing an initial decrease in HR and an increase in BRS in contrast to the hot drink. Consuming alcohol at a hot temperature seemed also to potentiate vascular effects as it induces early vasodilation and rapidly increases SkBf and hand temperature. The early changes with hot mixed wine may be of clinical relevance when associated with a meal, which in itself increases HR and promotes vasodilation. This could be particularly true in the elderly subjects who are prone to orthostatic and postprandial hypotension.

## Author Contributions

DS contributed to the study design, performed data acquisition and analysis, drafted the manuscript and contributed to interpretation of data, and critical revision of the work for important intellectual content. CM contributed to interpretation of the data, and critical revision of the manuscript for important intellectual content. J-PM conceived and designed the research, contributed to interpretation of data, and critical revision of the manuscript for important intellectual content.

## Conflict of Interest Statement

The authors declare that the research was conducted in the absence of any commercial or financial relationships that could be construed as a potential conflict of interest.

## Footnotes

1 https://www.randomizer.org/
